# Human papillomavirus ‘reflex’ testing as a screening method in cases of minor cytological abnormalities

**DOI:** 10.1038/sj.bjc.6604504

**Published:** 2008-08-05

**Authors:** M Fröberg, B Johansson, A Hjerpe, S Andersson

**Affiliations:** 1 Division of Obstetrics and Gynecology, Department of Clinical Science, Intervention and Technology, Karolinska University Hospital Huddinge, Karolinska Institutet, Stockholm 141 86, Sweden; 2 Division of Clinical Virology, Department of Laboratory Medicine, Karolinska University Hospital Huddinge, Karolinska Institutet, Stockholm 141 86, Sweden; 3 Division of Pathology, Department of Laboratory Medicine, Karolinska University Hospital Huddinge, Karolinska Institutet, Stockholm S-141 86, Sweden; 4 Centre for Clinical Research (CKF), Uppsala University, Centrallasarettet, Västerås 721 89, Sweden

**Keywords:** human papilloma virus (HPV), genotyping, liquid-based cytology (LBC), atypical squamous cells of undetermined significance (ASCUS), low-grade squamous intraepithelial lesion (LSIL), cervical intraepithelial neoplasia (CIN)

## Abstract

The aim was to evaluate human papillomavirus (HPV) ‘reflex genotyping’ in cases of minor cytological abnormalities detected in the gynaecological screening programme in Stockholm, Sweden. Liquid-based cytology samples showing minor cytological abnormalities were analysed using HPV genotyping (Linear Array, Roche diagnostics). Colposcopically directed cervical biopsies were obtained and the HPV test results were correlated with the histological results. In all, 63% (70/112) of the samples were high-risk (HR) HPV (HR-HPV) positive. A statistically significant correlation was found between high-grade cervical lesions and HR-HPV (*P*=0.019), among which HPV 16, 18, and 31 were the most important. The negative predictive value of HR-HPV detection for histologically confirmed high-grade lesions was 100%. An age limit for HPV reflex testing may be motivated in cases of low-grade squamous intraepithelial neoplasia (LSIL), because of high HR-HPV prevalence among younger women. By using HPV reflex genotyping, additional extensive workup can safely be avoided in about 50% of all cases of atypical squamous cells of undetermined significance (ASCUS) and LSIL among women ⩾30 years. This screening strategy could potentially reduce the total abnormal cytology-reporting rate in the Swedish screening programme by about 1% and provide more accurately directed follow-up, guided by cytological appearance and HPV test results.

Globally, cervical cancer is the second most common cancer, after breast cancer, among women (Kamangar *et al*, 2006). Human papillomavirus (HPV) infection is a prerequisite for development of cervical cancer (Bosch *et al*, 1995; Walboomers *et al*, 1999). A number of HPV types have been defined as high-risk (HR) HPV (HR-HPV) types, based on their association with cervical cancer (Muñoz *et al*, 2006). However, HPV infection is also common among healthy women. Generally, HPV infection is the most common sexually transmitted infection, and particularly common among young women (Bosch and de Sanjosé, 2007). It is usually asymptomatic and spontaneously cleared by the immune system (Franco *et al*, 1999).

In Sweden, significant decreases in the incidence and mortality of cervical cancer have been observed since the introduction of a population-based gynaecological screening programme in the 1960 s (Bergström *et al*, 1999; Gunnell *et al*, 2007). However, about 450 new cases of invasive cervical cancer are identified each year. The failure to prevent these cases is due to incomplete screening coverage, inadequate follow-up of abnormal smears, and methodological limitations in sensitivity for the detection of premalignant and malignant cervical lesions (Andersson-Ellström *et al*, 2000; Andersson *et al*, 2003; Andrae *et al*, 2008).

Liquid-based cytology (LBC) has shown higher sensitivity for high-grade cervical lesions than conventional cytology (Strander *et al*, 2007; Zhu *et al*, 2007). The proportion of abnormal findings increases when conventional Pap smears are replaced by LBC for screening. However, the positive predictive value (PPV) for advanced lesions increases in laboratories with extensive experience (Strander *et al*, 2007).

Today, in Sweden, about 3–5% of all smears show some kind of abnormality, almost 80% of which are minor cytological changes. The recommendations for the management of minor cytological abnormalities vary in different parts of Sweden. In Stockholm County, all abnormal cytology results lead to costly follow-up investigations with repeat Pap smear and colposcopically directed biopsies.

In cases of minor cytological abnormalities, HPV testing can more efficiently identify the small proportion of clinically important lesions. Such an approach is internationally accepted for cases of atypical squamous cells of undetermined significance (ASCUS), but has not been generally recommended in cases of low-grade squamous intraepithelial lesions (LSIL) because of a high prevalence of oncogenic HPV in this group (Scherman *et al*, 2002; Schiffman and Solomon, 2003; Arbyn *et al*, 2006; Wright *et al*, 2007).

Such triage can be performed in conjunction with LBC as a reflex test and may further improve cytological evaluation. The aim of this study was to determine the value of HPV genotyping as a ‘reflex test’ in cases of minor cytological abnormalities detected in the Swedish population-based primary screening programme.

## Materials and methods

### Study population and setting

Seven maternity health centres in the southern part of Stockholm participated in this study. During the study period (September 2005–September 2006), 4204 liquid-based consecutively obtained samples from routine screening were prepared, using the ThinPrep 2000 Processor (Cytyc Corporation, Boxborough, MA, USA) (Linder, 1998).

Minor cytological abnormalities were found in 149 (3.5%) of these samples, comprising the cytological classifications of ASCUS, atypical glandular cells of undetermined significance (AGUS), and LSIL.

The cytological diagnoses were defined using the Bethesda nomenclature (Solomon *et al*, 2002). According to Swedish recommendations, cases of koilocytosis without signs of dysplasia are reported as non-pathologic. Therefore, the LSIL group contains samples corresponding to cytological cervical intraepithelial neoplasia grade 1 (CIN1) only. There were 93 cases of LSIL, 53 of ASCUS, and 3 of AGUS. The two latter groups will hereafter be considered together and referred to as ASCUS.

Mean age of patients was 33 years, median age 30 years, and age range 22–59 years. Women with ASCUS were older than women with LSIL; mean ages of 35 years and 31 years, respectively (*P*=0.028), although the samples were consecutively derived from the same screening cohort. The age range was similar for both groups: 22–58 years for ASCUS and 22–59 years for LSIL.

Women with minor cytological abnormalities were referred for further investigation, including gynaecological examination, colposcopy, directed biopsies, and repeat Pap smear. The histological samples were evaluated and classified according to the CIN classification (Richard, 1968). On the basis of the most severe grade of CIN found, the histology findings were classified as within normal limits (WNL), CIN1, or CIN grade 2 or a more advanced lesion (CIN2+). The histological follow-up results were traced through the medical and laboratory records and through the Stockholm Oncology Center. Only histological material obtained within 1 year of cytological screening were registered. Ethical approval was obtained in December 2004 (No. 04-679/3).

### HPV analysis

Briefly, 2 ml of the remaining cell suspension from the liquid based cytology samples were taken for DNA extraction by a MagNA Pure LC Robot (Roche®). HPV-DNA detection and genotyping were performed using the Linear Array HPV Genotyping Test (LA) according to the manual provided by the manufacturer (Roche Diagnostics). Thus, HPV-DNA was amplified by PCR using a pool of biotin-labelled primers that hybridise in the L1 region. Then, HPV-DNA amplicons were chemically denatured to single-stranded DNA, hybridised with matching type-specific DNA probes, immobilized on nylon strips, and detected by colorimetric determination.

The 37 HPV types included in the LA test were divided into three ‘risk categories’: the HR-HPV types 16, 18, 31, 33, 35, 39, 45, 51, 52, 56, 58, and 59, the probably HR (pHR)-HPV types 26, 53, 66, 68, 73, and 82; and those considered to be low-risk (LR)-HPV or of undetermined risk including types 6, 11, 40, 42, 54, *55*, 61, *62*, 64, *67*, *69*, 70, *71*, 72, 81, *83*, *84*, IS39, and CP6108. Those of undetermined risk (shown in italics) will be considered together with the LR-HPV types (Muñoz *et al*, 2006).

The ability of the HPV test to identify or exclude a high-grade cervical lesion was evaluated with the histopathology as gold standard.

### Statistical analysis

To determine the statistical significance of differences between groups, *χ*^2^ statistics (exact tests), and Student's *t*-test were used. The null hypothesis of no difference was rejected at a significance level of *P*⩽0.05.

The primary end point was histopathologically verified CIN2+. The sensitivity, specificity, negative predictive value (NPV), and PPV of HR-HPV detection using LA to detect such high-grade lesions (CIN2+) were calculated, with 95% confidence intervals (CI; Newcombe, 1998).

## Results

### Histological findings

Of the 149 cases of mild cytological abnormalities studied, histopathological follow-up was obtained in 112 cases. Histology results were available for 39/56 (70%) of the ASCUS and 73/93 (78%) of the LSIL samples. These are the cases that will be discussed here later.

The cytological findings in relation to the histological diagnosis in 112 women are summarised in Table 1. Among women with minor cytological abnormalities, 39/112 (35%) CIN1 and 15/112 (13%) CIN2+ cases were detected. In cases reported as ASCUS, 11/39 (28%) CIN1 and 5/39 (13%) CIN2+ lesions were found. In cases of LSIL, there was a slightly higher detection rate of CIN1 (28/73 cases or 38%), but a similar rate of CIN2+ (10/73 cases or 14%).

### HPV infection and histological findings

In all, 81% (91/112) of all samples were HPV positive. There was a significant proportion of multiple infections and overlap of infections with HPV types from one, two, or all three risk categories (HR-, pHR-, and LR-HPV).

The prevalence of HPV-infected samples increased with the grade of dysplasia: 74% (43/58) of the WNL, 85% (33/39) of the CIN1, and 100% (15/15) of the CIN2+ cases were HPV positive (*P*=0.026).

**Figure 1:**
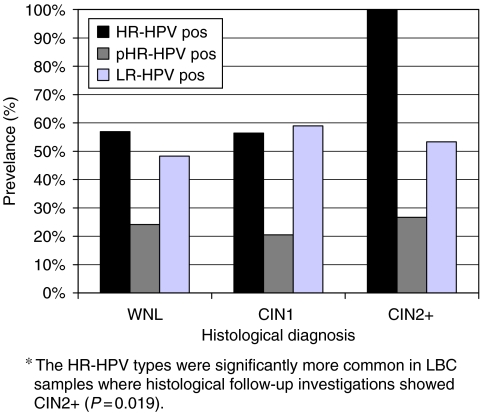
Prevalence of HPV risk categories related to histological diagnosis in 112 cases of minor cytological abnormalities. Single and multiple HPV infections are present within all risk categories. Since infection by HPV types of different risk categories overlap each other, the sum of the HR-, pHR-, and LR-HPV prevalences in each histological diagnosis group can exceed 100%. WNL (*n*=58)=within normal limits. CIN1 (*n*=39)=cervical intraepithelial neoplasia grade 1. CIN2+ (*n*=15): cervical intraepithelial neoplasia grade 2 or a more advanced lesion. HR-HPV pos (black bars)=samples positive for at least one HR-HPV type, with or without pHR- and/or LR-HPV coinfection. pHR-HPV pos (dark grey bars)=samples positive for at least one pHR-HPV type, with or without HR- and/or LR-HPV coinfection. LR-HPV pos (light grey bars)=samples positive for at least one LR-HPV type, with or without HR- and/or pHR-HPV coinfection.

Figure 1 demonstrates the prevalence of the different HPV risk categories, including cases of single and multiple HPV infections, with or without coinfections by HPV types belonging to other risk categories. All 15 cases of CIN2+ were HR-HPV positive (Figure 1) compared to 56% (22/39) of the CIN1 and 57% (33/58) of the WNL cases (*P*=0.019). In all, 63% (70/112) of the samples were HR-HPV positive (Table 2). In this cytologically selected material, the NPV for the LA-HPV test to detect a CIN2+ lesion was 100% (95% CI: 90–100%). The statistically significant relation between the HR-HPV group and CIN2+ disappears if the pHR-HPV types are considered as HR-HPV (*P*=0.112).

**Figure 2:**
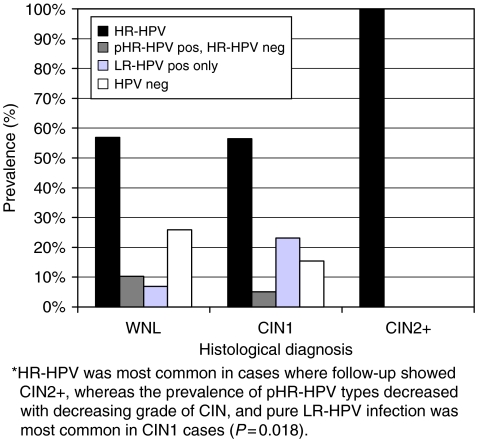
Prevalence of HPV hierarchic risk categories related to histological diagnosis in 112 cases of minor cytological abnormalities. The HPV test results have been classified into risk categories according to the HPV type/types of the highest HPV risk category found in each sample. Using this description of the HPV infection pattern, there is no overlap between the different HPV risk categories. HR-HPV pos (black bars)=samples positive for at least one HR-HPV type, with or without pHR- and/or LR-HPV coinfection. pHR-HPV pos, HR-HPV neg (dark grey bars)=samples positive for at least one pHR-HPV type but negative for HR-HPV, with or without LR-HPV coinfection. LR-HPV pos only (light grey bars)= samples positive for at least one LR-HPV type, without HR- and pHR-HPV coinfection. HPV neg (white bars)=samples negative for all detectable HPV types.

In Figure 2, the HPV infection pattern is described as ‘hierarchic’ risk categories; the HPV test results have been classified into risk categories according to the HPV type/types of the highest HPV risk category found in each sample. Pure LR-HPV infection was markedly overrepresented in CIN1, present in 23% of cases (9/39), compared with 7% (4/58) in the WNL cases, and the prevalence of pHR-HPV positive but HR-HPV negative cases decreased with increasing grade of CIN (*P*=0.018).

### Distribution of HPV types and histological findings

**Figure 3:**
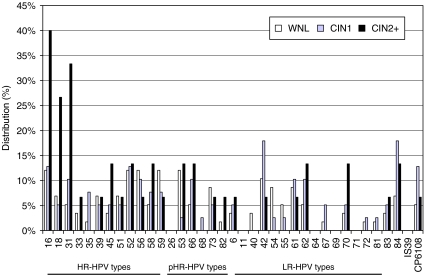
Relative distribution of HR-, pHR-, and LR-HPV types in liquid-based cytology samples showing minor cytological abnormalities, related to histological diagnosis: WNL (*n*=58; light grey bars), CIN1 (*n*=39; dark grey bars), and CIN2+ (*n*=15; black bars). The corresponding figures are presented in detail in Supplementary Table 1.

To detect all cases of CIN2+, the assay had to accurately detect at least the HPV types 16, 18, 31, 52, and 58. HPV 45 was detected as frequently as HPV 52 and 58 among the CIN2+ cases (2/15 cases or 13%); however, never as a single HR-HPV infection. In this limited material, we were able to demonstrate a significant correlation between HPV types 16 (*P*=0.045), 18 (*P*=0.039), and 31 (*P*=0.006) with high-grade cervical lesions. Presence of coinfection with other HPV types has not been taken into consideration when performing the calculations (Figure 3, Supplementary Table 1). Among patients with minor cytological lesions and subsequent histological findings of CIN2+, the HPV types 16, 31, and 18 clearly stand out as the most common, present in single or multiple HPV infections in 40% (6/15), 33% (5/15), and 27% (4/15) of the cases, respectively. Together, they were present in 73% (11/15) of the samples. Out of the 15 CIN2+ cases, 53% (8/15) were infected with HPV 16 and/or HPV 18; however, only three of these cases were not infected with additional HR-HPV types.

In the CIN1 and WNL cases, the HPV types were more evenly distributed. Among the cases of CIN1, there was a relatively high proportion of the LR-HPV types HPV 42, 84, and CP6108. HPV 6 was present in only 4% (5/112) of all cases, and there were no HPV 11 infections.

### Multiple HPV infection and degree of CIN

**Figure 4:**
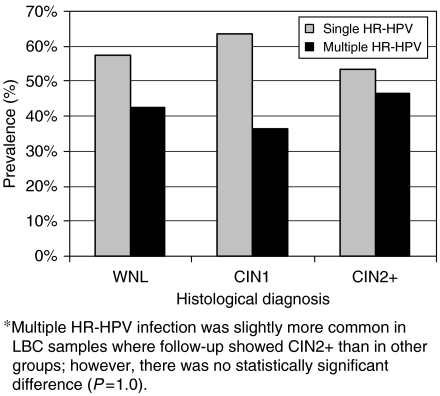
Prevalence of single *vs* multiple HR-HPV infections in HR-HPV-positive cases related to histological diagnosis: WNL (*n*=33), CIN1 (*n*=22), and CIN2+ (*n*=15). Single HR-HPV infections (light grey bars). Multiple HR-HPV infections (black bars).

In all, 54% (61/112) of the cases had multiple HPV infection, and of all 91 HPV-infected individuals, 67% (61/91) were infected by more than one HPV type. Multiple HPV infection was slightly more common in cases of histological CIN2+ than in CIN1 and WNL cases (73, 64, and 67%, respectively). Among the HR-HPV-positive samples, 41% (29/70) were infected by more than one HR-HPV type. Also in the case of HR-HPV, multiple infections were slightly more common in cases of CIN2+ (Figure 4) than in CIN1 and WNL cases (47 *vs* 36, and 42%, respectively). However, none of these differences were statistically significant, but of course, a multiple HPV infection increases the probability of harbouring a highly oncogenic HPV type.

## Discussion

In the Swedish population-based gynaecological screening programme, abnormal samples are associated with follow-up investigations, and clinical recommendations for the management of minor cytological abnormalities vary. In Stockholm County, all women with any degree of atypia are referred for a colposcopic examination and targeting biopsy. The question of whether HPV testing may improve the Swedish screening programme is under discussion, especially for use as a follow-up test to identify possible precancerous lesions among mild cytological abnormalities and distinguish these from reactive and degenerative lesions. This is an approach, which is widely accepted internationally (see introduction), but has only been randomly adopted in Sweden.

Reporting rates in the Scandinavian screening programmes differ from those in the Anglo-Saxon world. Scandinavian cytologists tend to rate the samples as WNL more frequently than British or American cytologists (abnormal smear rate: 3–5 *vs* 10–15%), discounting subtle changes in deference to achieving cytological certainty (Scott *et al*, 2002). In spite of these differences, our results suggest that the value of HR-HPV detection in cases of minor cytological abnormalities is high also in a Swedish context.

Among cases of minor cytological abnormalities detected using LBC, HR-HPV detection using LA has a 100% (95% CI: 90–100%) NPV for high-grade cervical dysplasia during a maximum follow-up time of 1 year (Table 2). This is similar to the findings in a large scale prospective study including cases of ASCUS where carcinogenic HPV detection using the same HPV test showed an excellent NPV (95.67%; 95% CI: 94.22–96.85%) for 2-year cumulative CIN2+ (Castle *et al*, 2008). In this study, we used a different definition for carcinogenic or HR-HPV, classifying HPV 66 and 68 as pHR-HPV types. This probably affects the performance of the HPV test only marginally. Our results suggest that the pHR-HPV group is of little of no importance in a screening context; however, this might need further consideration.

In the LSIL group, HR-HPV prevalence is high, about 80–85%, among women younger than 30 years, and drops to about 50% among women aged 30 years, or older. For this reason, specificity of HPV detection test can be improved by setting an age limit of 30 years for HPV reflex testing in cases of LSIL (Table 2). An age limit at 35 years in cases of LSIL has been suggested (Ronco *et al*, 2007), yielding a somewhat higher specificity for histologically confirmed CIN2+ (using Hybrid Capture II for HPV detection), compared to our findings for women aged 30–59 years.

In cases of ASCUS, where HR-HPV prevalence is considerably lower (about 40%), and the HR-HPV prevalence was not significantly higher among women under the age of 30 years than among older women, for which reason the diagnostic value of HPV testing in this group is independent of age.

Thus, in HPV triage using the LA-HPV test for all cases of ASCUS and for cases of LSIL with age ⩾30 years, the need for extensive follow-up investigations would be reduced by about 50%, with good safety. In such cases, cytological evaluation and virological analysis could be performed using the same material (reflex testing). Because there is only one sampling event, sampling error would be reduced. Screening programme costs would also be reduced, because both analyses can be performed without recalling the patient. The decision of whether to set an age limit for HPV reflex testing in LSIL cases should be based on health–economic calculations.

According to our results, only the HR-HPV group had a statistically significant correlation with CIN2+, for which the HR-HPV types 31, 18, and 16 were the most important (Figure 3, Supplementary Table 1). In this small material, in order to identify all cases of high-grade cervical lesions, the HPV test had to accurately detect HPV 16, 18, 31, 52, and 58. These HPV types are represented among the main worldwide oncogenic HPV types (HPV 16, 18, 31, 33, 35, 45, 52, and 58), which account for about 88% of all cervical cancer cases worldwide (Smiths *et al*, 2007). However, since a high NPV is necessary in a reflex screening situation, all HR-HPV types need to be accurately detected.

In cases where histology findings were WNL or CIN1, more than 50% were HR-HPV positive. These women need to be followed up, given their elevated risk for developing a high-grade cervical lesion. Repeated HPV genotyping allows diagnosing persistent HPV infection and risk stratification of women with cytological abnormalities, and might be useful for clinical management (Wheeler *et al*, 2006; Bulkmans *et al*, 2007; Naucler *et al*, 2007). However, the exact design of such follow-up needs to be studied further.

Liquid-based cytology (LBC) combined with reflex detection of HR-HPV types in cases of minor cytological abnormalities probably provides increased sensitivity and increased specificity for the detection of high-grade cervical lesions, when used in population-based screening.

Based on the fact that minor cytological abnormalities constitute 75–80% of all abnormal cytological results in Sweden, our preliminary calculations show that combining LBC with HPV reflex testing in gynaecological screening reduces the total abnormal cytology-reporting rate by approximately 1%. However, LBC screening generally yields a somewhat higher abnormal cytology-reporting rate than conventional cytology screening. Conventional cytology is the predominating screening method in Sweden today, which makes the potential 1% reduction of the abnormal cytology-reporting rate uncertain. The main value of introducing LBC combined with HPV reflex testing is probably an improved ability to identify women with an increased risk of developing premalignant and malignant cervical lesions, without increasing the abnormal cytology-reporting rate. A cost–efficiency analysis is ongoing. Unnecessary investigations and unnecessary psychological stress could be avoided, and resources could be used for more accurately directed follow-up of women with abnormal cytological findings.

## Figures and Tables

**Table 1:** Liquid-based cytology findings correlated to histological diagnosis in 112 women with minor cytological abnormalities

	**Histological diagnosis**			
**Cytology**	**WNL (%)**	**CIN1 (%)**	**CIN2+ (%)**	**Total (%)**
ASCUS	23 (59)	11 (28)	5 (13)	39 (100)
LSIL	35 (48)	28 (38)	10 (14)	73 (100)
Total	58 (52)	39 (35)	15 (13)	112 (100)

ASCUS=atypical squamous cells of undetermined significance; CIN1=cervical intraepithelial neoplasia grade 1; CIN2+=cervical intraepithelial neoplasia grade 2 or a more advanced lesion; LSIL=low-grade squamous intraepithelial lesion; WNL=within normal limits.

The differences in the detection of histological CIN1 and CIN2+ were not statistically significant (*P*=0.410).

**Table 2:** Performance of HR-HPV detection using linear array to detect histologically confirmed CIN2+ in cases of ASCUS and LSIL, with respect to sensitivity, specificity, positive predictive value (PPV), and negative predictive value (NPV) with 95% confidence intervals (CI)

**Cytology classification**	**Probability of HR-HPV presence (%) (95% CI)**	**Sensitivity (%) (95% CI)**	**Specificity (%) (95% CI)**	**PPV (%) (95% CI)**	**NPV (%) (95% CI)**
ASCUS+LSIL (*n*=112)	63 (53–71)	100 (75–100)	43 (33–54)	21 (13–33)	100 (90–100)
ASCUS (*n*=39)	46 (30–63)	100 (46–100)	62 (44–77)	28 (11–54)	100 (81–100)
LSIL (*n*=73)	71 (59–81)	100 (66–100)	33 (22–46)	19 (10–33)	100 (81–100)
LSIL ⩾30 yrs (*n*=32)	53 (35–70)	100 (40–100)	54 (34–72)	24 (8–50)	100 (75–100)
ASCUS+LSIL ⩾30 years (*n*=71)	49 (37–61)	100 (63–100)	58 (45–70)	26 (13–44)	100 (88–100)

ASCUS=atypical squamous cells of undetermined significance; CIN2+=cervical intraepithelial neoplasia grade 2 or a more advanced lesion; HPV=human papilloma virus; HR−HPV=high−risk HPV; LSIL=low−grade squamous intraepithelial lesion; WNL=within normal limits.
