# Supplementary material for: Human papillomavirus ‘reflex’ testing as a screening method in cases of minor cytological abnormalities
Source: Br J Cancer. 2008 Aug 5;99(4):563–8. doi: 10.1038/sj.bjc.6604504 (PMC2527833; doi:10.1038/sj.bjc.6604504)
Supplement: Supplementary Table 1 [file 6604504x1.doc]

**Supplementary Table 1.** Relative distribution of HR-, pHR- and LR-HPV-types in liquid-based cytology samples showing minor abnormalities, related to histological diagnosis**.** The table is a supplement to Figure 3.

| HPV type | WNL (n=58) | CIN1 (n=39) | CIN2+ (n=15) | Total  (n=112) | p-value |
| --- | --- | --- | --- | --- | --- |
| HR-HPV types |  |  |  |  |  |
| 16 | 7 (12%) | 5 (13%) | 6 (40%) | 18 (16%) | 0.045 |
| 18 | 4 (7%) | 2 (5%) | 4 (27%) | 10 (9%) | 0.038 |
| 31 | 3 (5%) | 4 (10%) | 5 (33%) | 12 (11%) | 0.006 |
| 33 | 2 (3%) | 0 | 1 (7%) | 3 (3%) | 1 |
| 35 | 1 (2%) | 3 (8%) | 0 | 4 (4%) | 0.728 |
| 39 | 4 (7%) | 2 (5%) | 1 (7%) | 7 (6%) | 1 |
| 45 | 2 (3%) | 2 (5%) | 2 (13%) | 6 (5%) | 0.238 |
| 51 | 4 (7%) | 2 (5%) | 1 (7%) | 7 (6%) | 1 |
| 52 | 7 (12%) | 5 (13%) | 2 (13%) | 14 (13%) | 1 |
| 56 | 7 (12%) | 4 (10%) | 1 (7%) | 12 (11%) | 0.921 |
| 58 | 3 (5%) | 3 (8%) | 2 (13%) | 8 (7%) | 0.306 |
| 59 | 7 (12%) | 3 (8%) | 1 (7%) | 11 (10%) | 0.680 |
| pHR-HPV |  |  |  |  |  |
| 26 | 0 | 0 | 0 | 0 | - |
| 53 | 7 (12%) | 1 (3%) | 2 (13%) | 10 (9%) | 0.235 |
| 66 | 3 (5%) | 4 (10%) | 2 (13%) | 9 (8%) | 0.570 |
| 68 | 0 | 1 (3%) | 0 | 1 (1%) | 0.475 |
| 73 | 5 (9%) | 2 (5%) | 1 (7%) | 8 (7%) | 0.878 |
| 82 | 1 (2%) | 0 | 1 (7%) | 2 (2%) | 0.636 |
| LR-HPV |  |  |  |  |  |
| 6 | 2 (3%) | 2 (5%) | 1 (7%) | 5 (4%) | 1 |
| 11 | 0 | 0 | 0 | 0 | - |
| 40 | 2 (3%) | 0 | 0 | 2 (2%) | 0.639 |
| 42 | 6 (10%) | 7 (18%) | 1 (7%) | 14 (13%) | 0.446 |
| 54 | 5 (9%) | 1 (3%) | 0 | 6 (5%) | 0.302 |
| 55 | 3 (5%) | 1 (3%) | 0 | 4 (4%) | 0.647 |
| 61 | 5 (9%) | 4 (10%) | 1 (7%) | 11 (10%) | 1 |
| 62 | 3 (5%) | 4 (10%) | 2 (13%) | 9 (8%) | 0.570 |
| 64 | 0 | 0 | 0 | 0 | - |
| 67 | 1 (2%) | 2 (5%) | 0 | 3 (3%) | 0.568 |
| 69 | 0 | 0 | 0 | 0 | - |
| 70 | 2 (3%) | 2 (5%) | 2 (13%) | 6 (5%) | 0.287 |
| 71 | 0 | 0 | 0 | 0 | - |
| 72 | 1 (2%) | 1 (3%) | 0 | 2 (2%) | 1 |
| 81 | 1 (2%) | 1 (3%) | 0 | 2 (2%) | 1 |
| 83 | 2 (3%) | 2 (5%) | 1 (7%) | 5 (4%) | 1 |
| 84 | 4 (7%) | 7 (18%) | 2 (13%) | 13 (12%) | 0.270 |
| IS39 | 0 | 0 | 0 | 0 | - |
| CP6108 | 3 (5%) | 5 (13%) | 1 (7%) | 9 (8%) | 0.385 |
